# Acute Myeloid Leukemia with Philadelphia Chromosome, Near-tetraploidy, and 5q Deletion

**DOI:** 10.7759/cureus.5606

**Published:** 2019-09-09

**Authors:** Abdul Moiz Khan, Ayesha Munir, Roshan Asrani, Saleh Najjar

**Affiliations:** 1 Internal Medicine, Albany Medical Center, Albany, USA; 2 Pathology, Albany Medical Center, Albany, USA

**Keywords:** acute myeloid leukemia, philadelphia chromosome, tyrosine kinase inhibitors, leukemia, hematology

## Abstract

A 49-year-old male presented to his physician with three weeks of dyspnea, dry cough, and fever. He did not respond to antibiotics and corticosteroids. He presented to the emergency department with worsening symptoms, where blood work revealed severe anemia, leukocytosis, thrombocytopenia, and 61% blasts on peripheral smear. Bone marrow biopsy showed acute myeloid leukemia (AML). While the results of other studies were awaited, treatment was begun with 7+3 induction (cytarabine and daunorubicin). Karyotyping returned positive for the BCR-ABL1 fusion gene (Philadelphia chromosome), near-tetraploidy, and 5q deletion. Follow-up bone marrow biopsy revealed residual disease (12% blasts). Re-induction was initiated with 5+2 cytarabine and daunorubicin with the addition of dasatinib. Subsequent bone marrow biopsies revealed minimal residual disease and BCR-ABL on polymerase chain reaction (PCR). The patient was placed on dasatinib maintenance and later switched to nilotinib. This case demonstrates the simultaneous presence of rare cytogenetic abnormalities in AML. It also discusses the successful utilization of tyrosine kinase inhibitors (TKIs) in the treatment of BCR-ABL-positive AML, as there are no established guidelines.

## Introduction

Cytogenetic analysis has become the cornerstone of diagnosis, treatment, and prognostication in various malignancies. BCR-ABL rearrangement corresponding to t(9;22)(q34;q11) translocation, commonly known as Philadelphia chromosome, is a hallmark of chronic myeloid leukemia (CML) and frequently associated with acute lymphoblastic leukemia (ALL) [1]. However, acute myeloid leukemia (AML) with BCR-ABL is rare and is reported to be around 0.5%-3% of all AML cases [1-4]. It was not until the World Health Organization (WHO) classification in 2016 that AML with the BCR-ABL1 fusion gene was finally accepted as a separate provisional entity [5-6]. Tetraploidy (4n, 92 chromosomes) and near-tetraploidy (81-103 chromosomes) are karyotype abnormalities described in certain hematological as well as solid malignancies. However, they are exceedingly uncommon in AML [7-8]. 5q deletion is the most common cytogenetic abnormality present in myelodysplastic syndrome (MDS) and may also be present in high-risk AML [6]. To date, there are no established guidelines on BCR-ABL-positive AML treatment. We present a case of AML with the simultaneous presence of the Philadelphia chromosome, near-tetraploidy, and 5q deletion. We will also discuss the treatment strategies as well as the clinical and molecular aspects of the disease.

## Case presentation

A 49-year-old male with no significant past medical or family history presented to his primary care physician with three weeks of dry cough, exertional dyspnea, low-grade fever, night sweats, fatigue, and generalized weakness. He was initially treated empirically with azithromycin and prednisone but failed to respond. He subsequently presented to the emergency department for a re-evaluation of worsening symptoms. Physical examination was significant for tachycardia, tachypnea, and pallor. Complete blood count showed severe anemia with significant leukocytosis and thrombocytopenia. Peripheral blood smear showed 61% blasts. Routine infectious workup, including chest X-ray, blood culture, sputum gram stain, and culture were negative. These findings prompted workup for hematological malignancy.

**Figure 1:**
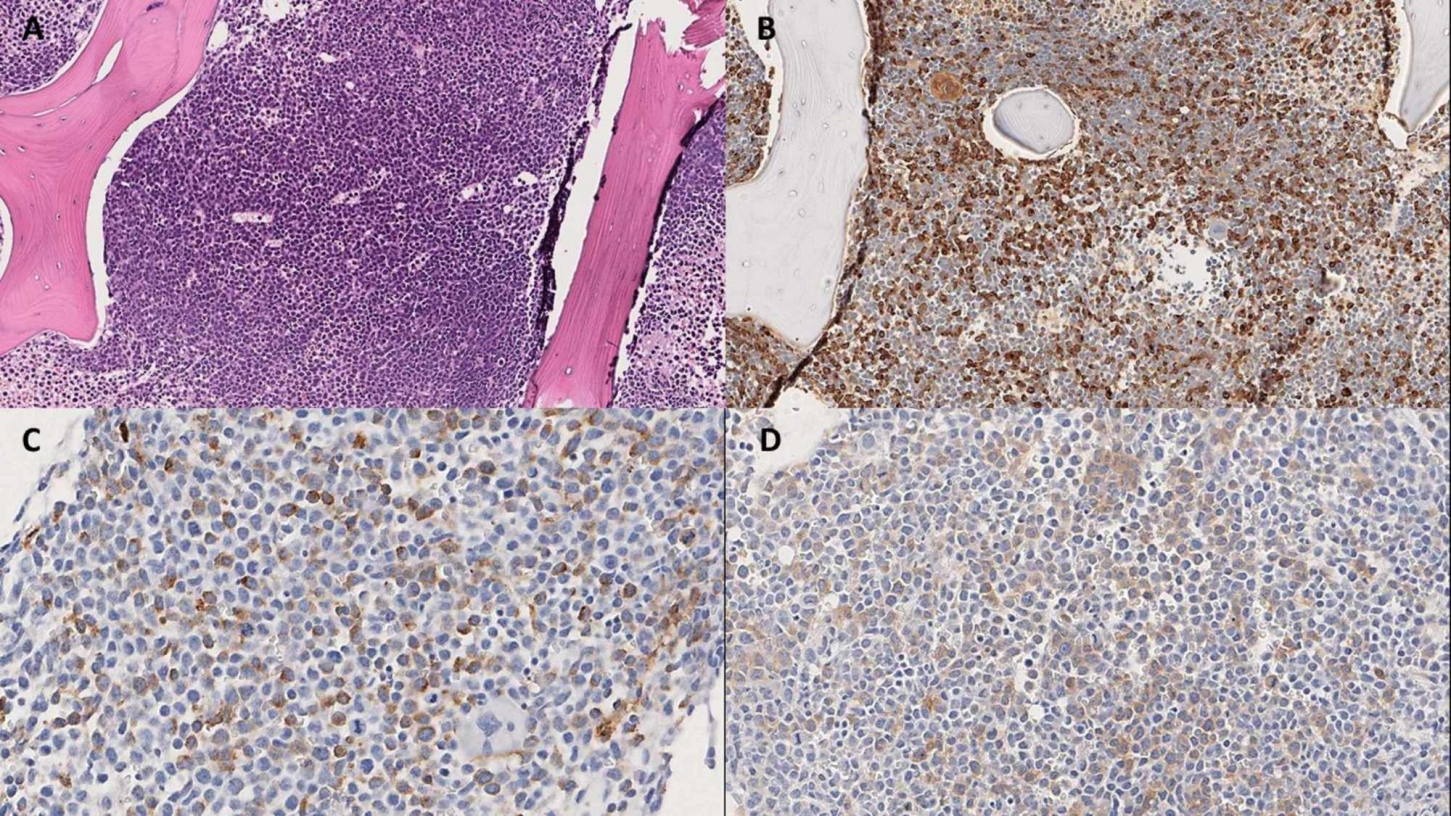
Bone marrow biopsy (A) H&E staining showing hypercellular marrow almost entirely replaced by the blasts. Immunohistochemistry with blasts staining for (B) lysozyme (C) CD 34 and (D) c-kit

The results of the initial workup are as follows: *Complete blood count (CBC) on presentation: White blood cells (WBCs) 33,300/μl, hemoglobin 6.5 g/dl, hematocrit 19.9%, platelets 85,000/μl *LDH: 888 units/L *Peripheral blood smear: Anemia with aniso-poikilocytosis, reduced platelets, increased WBCs with 61% blasts *Flow cytometry of peripheral blood: 63% large blasts positive for CD117, HLA-DR, CD34, CD13, TdT, and CD45 (moderate); partial expression of CD33, CD22, and CD11b; negative for CD14, CD56, MPO, CD19, CD20, surface light chains, CD3, CD4, CD5, and CD7; immunophenotype most consistent with AML. *Initial bone marrow biopsy findings (Figure 1): Hypercellular marrow 80%-90%, almost entirely replaced by blasts.

While detailed results were awaited, based on the very high percentage of myeloblasts in the peripheral smear and preliminary findings of bone marrow biopsy, the patient was started on standard 7+3 induction chemotherapy with cytarabine and daunorubicin.

The results of the remaining workup were: *Bone marrow flow cytometry: 70% large blasts positive for CD117, HLA-DR, CD34, CD13, CD33, TdT, and CD45 (moderate); partial expression of CD22 and CD11b; largely negative for CD14, CD56, CD19, CD20, CD3, CD4, CD5, and CD7 22%; immunophenotype most consistent with AML. *Immunohistochemistry: Blasts positive for CD34, lysozyme, CD117, and myeloperoxidase (MPO). *Karyotyping/chromosome analysis: “95~98, XXYY, del(5)(q31q35), +6, t(9;22)(q34;q11.2)x2, -16, +17, +19, +22, +22, +der(22)t(9;22)(q34;q11.2)[cp18]/46,XY(2); abnormal karyotype, male.” *Eighteen of the 20 mitotic cells were near-tetraploid, had loss of one copy of chromosome 16, an additional copy of chromosomes 6, 17, and 19, two additional copies of chromosome 22, a deletion in the long arm of chromosome 5, two copies of translocation between the long arms of chromosomes 9 and 22, resulting in two Philadelphia (Ph) chromosomes, and an additional Philadelphia (Ph) chromosome. *Mutation analysis: Negative for NPM1, IDH1/IDH2, c-KIT, CEBPA, FLT3 ITD/TKD mutation. *Fluorescence in-situ hybridization (FISH): Trisomy, tetrasomy, and pentasomy of chromosome 7, 8, and 20 consistent with a polyploid neoplastic clone; negative for RUNX1T1/RUNX1 (ETO/AML1), KMT2A (MLL), PML/RARA, CBFB rearrangements; negative for monosomy 5 and 7, trisomy 8, 7q deletion, and PTPRT deletion.

Bone marrow biopsy on Day 21 revealed residual disease with 12% blasts and the biochemical presence of BCR-ABL on quantitative PCR (0.5406%). The patient underwent re-induction with 5+2 cytarabine and daunorubicin, this time with the addition of dasatinib (BCR-ABL tyrosine kinase inhibitor). He was continued on prophylactic allopurinol, acyclovir, and voriconazole. Follow-up bone marrow biopsy was negative for any morphological evidence of blasts but quantitative PCR for BCR-ABL was positive (.0745%), indicating minimal residual disease (MRD). The patient was placed on oral dasatinib maintenance therapy and later switched to nilotinib. This was because our patient had developed a parapneumonic effusion but it was decided to avoid a drug like dasatinib, which may cause pleural effusions as an adverse effect.

This case report encompasses a period of eight months from the time of diagnosis. For AML, given the presence of MRD, as demonstrated by positive quantitative BCR-ABL, the patient will be continued indefinitely on daily nilotinib maintenance therapy. This will be followed by quantitative serum PCR for BCR-ABL every four weeks and bone marrow biopsy every 12 weeks or earlier if clinically warranted. Of note, the patient developed necrotizing pneumonia with empyema during the course of treatment, which was treated with antibiotics and chest tube placement. The empyema recurred, therefore, thoracoscopic decortication was done later. He is not currently a candidate for allogeneic stem cell transplantation (ASCT), given the clinical condition and frailty. However, it may be pursued once he recovers from the pulmonary complications.

## Discussion

According to the 2017 European Leukemia Net (ELN) risk stratification by genetics, BCR-ABL positivity, complex karyotype (like near-tetraploidy), and 5q deletion are all placed in the ‘adverse’ risk category [9]. The presence of BCR-ABL not only has prognostic implications but also offers the potential for targeted therapy. Tyrosine kinase inhibitors (TKIs) like imatinib and dasatinib are the standard of care in CML, but no guidelines have been established regarding their use in Philadelphia chromosome-positive AML.

AML with Philadelphia chromosome poses a challenge in distinguishing it from CML with blast crisis. As in our patient, the absence of any antecedent hematological abnormality and lack of basophilia or splenomegaly are clues that favor de-novo AML versus CML transformation.

There has been some improvement in the prognosis with the use of TKIs, increasing the median overall survival from nine months to 18 months [4]. Isolated case reports yield promising results with the use of imatinib in induction with concomitant standard chemotherapy, as well as in consolidation and continuous maintenance therapy [4,10-11]. TKIs have been effectively used as a bridge to definitive allogeneic stem-cell transplantation (ASCT) [12-13]. Nevertheless, TKIs alone have not demonstrated sustained responses except in rare cases [14]. This may be due to the rapid clonal evolution and development of resistance to TKIs [1]. Second, while BCR-ABL may induce a proliferative advantage in AML, unlike in CML, it is not likely the primary driver mutation [1]. Therefore, age and risk-factor-appropriate intensive induction chemotherapy followed by consolidation with chemotherapy or allogeneic stem cell transplantation (ASCT) should still serve as the mainstay of treatment [1,11,15]. In our patient, we were able to achieve and maintain MRD by utilizing dasatinib during re-induction followed by dasatinib or nilotinib monotherapy for maintenance.

5q deletion is the most frequent cytogenetic aberration in MDS and can be seen in high-risk AML [6]. Two distinct regions of deletion are frequent: 5q31 and 5q33. Most deletions involve both loci-like del(5)(q31q35) seen in our patient [16]. Genes implicated in these deletions include tumor suppressor genes like CTNNA1, EGR1, and CD25C, antiangiogenic and antiproliferative genes like SPARC, micro-RNA genes, and ribosomal protein genes like RPS14, which may employ p53 dependent and independent mechanisms [16-17].

Tetraploidy and near-tetraploidy (T/NT) is a rare finding in AML occurring in 0.7%-1.2% of adult AML patients [7]. Patients are typically older males [7-8]. Although NT/T confers an adverse risk, it is most often found concurrently with other cytogenetic abnormalities, thus making it hard to quantify its isolated impact [18]. This is the case in our patient as well, where other cytogenetic abnormalities are dictating not only the course of the disease but the treatment choices as well.

## Conclusions

Although the BCR-ABL fusion gene is pathognomonic for CML and frequently associated with ALL, it may also play a role in the treatment course of AML. TKIs are effective adjuncts for treating BCR-ABL positive AML. However, monotherapy with TKIs is not substantiated for induction or consolidation. Therefore, age and risk factor-appropriate chemotherapy and ASCT should still be used as the first-line treatment until more robust guidelines are created. TKIs may be useful as single agents in continuous maintenance therapy and as a bridge to other first-line therapies in AML such as ASCT.
